# Alzheimer’s disease and cigarette smoke components: effects of nicotine, PAHs, and Cd(II), Cr(III), Pb(II), Pb(IV) ions on amyloid-β peptide aggregation

**DOI:** 10.1038/s41598-017-13759-5

**Published:** 2017-10-31

**Authors:** Cecilia Wallin, Sabrina B. Sholts, Nicklas Österlund, Jinghui Luo, Jüri Jarvet, Per M. Roos, Leopold Ilag, Astrid Gräslund, Sebastian K. T. S. Wärmländer

**Affiliations:** 1 Department of Biochemistry and Biophysics, Arrhenius Laboratories, Stockholm University, 106 91 Stockholm, Sweden; 2 Department of Anthropology, National Museum of Natural History, Smithsonian Institution, Washington, DC USA; 3 Department of Environmental Science and Analytical Chemistry, Arrhenius Laboratories, Stockholm University, 106 91 Stockholm, Sweden; 4 Chemical Research Laboratory, University of Oxford, 12 Mansfield Road, Oxford Ox, 1 3TA UK; 5 The National Institute of Chemical Physics and Biophysics, Tallinn, Estonia; 6 Institute of Environmental Medicine, Karolinska Institutet, Nobels väg 13, 171 77 Stockholm, Sweden; 7 Department of Clinical Physiology, Capio St.Göran Hospital, St.Göransplan 1, 112 19 Stockholm, Sweden

## Abstract

Cigarette smoking is a significant risk factor for Alzheimer’s disease (AD), which is associated with extracellular brain deposits of amyloid plaques containing aggregated amyloid-β (Aβ) peptides. Aβ aggregation occurs via multiple pathways that can be influenced by various compounds. Here, we used AFM imaging and NMR, fluorescence, and mass spectrometry to monitor *in vitro* how Aβ aggregation is affected by the cigarette-related compounds nicotine, polycyclic aromatic hydrocarbons (PAHs) with one to five aromatic rings, and the metal ions Cd(II), Cr(III), Pb(II), and Pb(IV). All PAHs and metal ions modulated the Aβ aggregation process. Cd(II), Cr(III), and Pb(II) ions displayed general electrostatic interactions with Aβ, whereas Pb(IV) ions showed specific transient binding coordination to the N-terminal Aβ segment. Thus, Pb(IV) ions are especially prone to interact with Aβ and affect its aggregation. While Pb(IV) ions affected mainly Aβ dimer and trimer formation, hydrophobic toluene mainly affected formation of larger aggregates such as tetramers. The uncharged and hydrophilic nicotine molecule showed no direct interactions with Aβ, nor did it affect Aβ aggregation. Our Aβ interaction results suggest a molecular rationale for the higher AD prevalence among smokers, and indicate that certain forms of lead in particular may constitute an environmental risk factor for AD.

## Introduction

Alzheimer’s disease (AD) is a progressive, irreversible, and currently incurable neurodegenerative disorder characterized by neuronal loss, memory impairment, and declining cognitive functions. As the leading cause of dementia in a rapidly aging population, AD is a growing threat to global health, economy, and society^1,2^. The worldwide prevalence of AD is predicted to quadruple in the 21^st^ century, thus affecting one in 85 people by 2050^2,3^. To potentially reduce the global burden of a looming AD epidemic, it is crucial to identify modifiable risk factors at the onset and/or early progression of the disease^3,4^.

**Figure 1:**
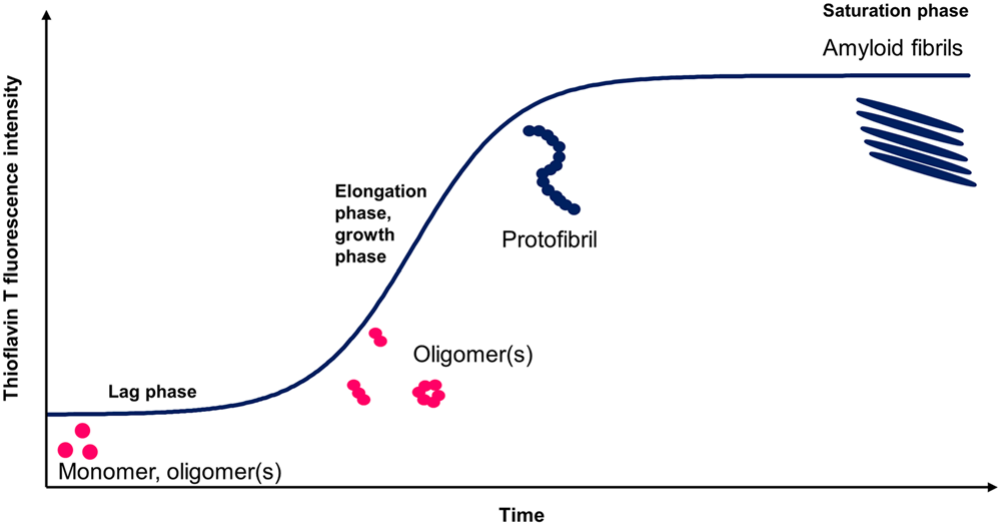
Simplified overview of the aggregation pathway for the amyloid-β peptide. Thioflavin T (ThT) fluorescence spectroscopy can be used to monitor the self-assembly of Aβ peptides from soluble monomers and oligomers into amyloid fibrils via various intermediate states such as protofibrils. The sigmoidal aggregation curve consists of a lag phase, or nucleation phase, and the elongation phase, or growth phase, before the saturation phase is reached. Monomeric and oligomeric conformational changes and nuclei formation processes occurs in the lag phase, followed by a rapid elongation process into partly insoluble fibrils. Aggregation modulation effects can be studied by monitoring the ThT fluorescence when Aβ is incubated with various compounds.

Because AD is difficult to detect and diagnose in its early stages^5^, many studies have focused on the etiology of late stage brain lesions. The characteristic AD lesion, first observed in human brain tissue in 1906, is extracellular amyloid plaques consisting mainly of amyloid-β (Aβ) peptides aggregated into insoluble fibrils^6^. Originally thought to be toxic, these plaques are now usually considered to be less harmful end products of an aggregation process involving formation of intermediate Aβ oligomers that appear to be neurotoxic (the so-called amyloid cascade hypothesis)^7–13^. The aggregation scheme of the Aβ peptide (Fig. 1) can be monitored with various experimental techniques and interaction agents^14^, including the fluorescent dye Thioflavin T (ThT) that displays increased fluorescence intensity when bound to amyloid aggregates^15^. In addition to amyloid plaques, AD brain tissue typically exhibits a second type of lesion in the form of intracellular neurofibrillary tangles consisting of aggregated hyperphosphorylated tau proteins. Exactly how Aβ or tau aggregation can induce the neuronal death associated with AD remains a point of contention. Different mechanisms for development of cell toxicity have been proposed that need not be mutually exclusive^16^. Although only around five percent of all AD cases are caused by inherited genetic conditions (i.e., the familial form), the higher incidence of AD among patients with aggregation-enhancing Aβ mutations provides an irrefutable link between AD and Aβ aggregation^17^.

Advanced age is a major risk factor for the more common sporadic AD, but environmental factors such as life style (e.g., diet, alcohol consumption, physical and mental exercise)^18–20^ and air pollution^21–24^ contribute to the disease as well^25^. While early studies were contradictory, there is now general consensus that cigarette smoking increases AD risk when factors such as survival bias, competing risk, and tobacco industry affiliation of the researchers have been taken into account^26–35^. Other neurodegenerative diseases such as amyotrophic lateral sclerosis (ALS)^36–39^, multiple sclerosis (MS)^40^ and Parkinson’s disease^41^ also appear to be more prevalent among smokers, although the causes underlying these increased risks remain unclear. Particularly debated are the possible neuroprotective effects of the parasympathomimetic stimulant nicotine, extracted from the tobacco plant (*Nicotiana tabacum*)^42–46^.

In addition to nicotine, tobacco contains high levels of various metals. In fact, the tobacco plant is so effective at extracting metals from the ground that it is sometimes used for phytoremediation of metal-contaminated soil and groundwater^47,48^. The metal content in tobacco leaves is further increased by the use of metal-containing fertilizers, herbicides, pesticides, and insecticides such as lead arsenate when growing commercial tobacco, and by the metal boxes used for growing, drying, and curing tobacco^49^. With additional contributions from other sources such as brightening agents in rolling paper, cigarettes end up containing non-negligible concentrations of metals such as Al, As, Cd, Co, Cr, Cu, Hg, Mn, Ni, Pb, Se, Tl, V, and Zn^47,50–53^, many of which (i.e., Al, As, Cd, Cu, Cu, Hg, Mn, Ni, Pb, Tl and V) are considered neurotoxic^54^. Numerous studies link AD to the essential transition metals Cu, Fe, and Zn, as they are elevated in phosphorylated tau tangles^55^ and in AD plaques compared to the surrounding nerve tissue (i.e., AD neuropil)^56–58^. Cu(II), Fe(II), and Zn(II) ions display specific binding to the Aβ peptide and modulate its aggregation pathways^59–64^, and Cu exposure increases Aβ levels in mice^65,66^. Cu(II) and Fe(III) ions furthermore generate harmful reactive oxygen species (ROS) when bound to the Aβ peptide^64^, and such ROS damage likely contributes to the neuroinflammatory condition associated with AD^67^. The previously suggested connections between AD and metals such as Al^68,69^ and Hg^70^ remain unclear but possibly valid. Although metal ion interactions and general metal dyshomeostasis appears to be an integral part of AD pathology^59,71^, studies on non-essential metals in AD at a molecular level are rare^72–74^.

Cigarette smoke contains thousands of organic compounds that are either present in the tobacco plant, added during cigarette manufacture, or produced by pyrolysis during smoking. The latter group includes polycyclic aromatic hydrocarbons (PAHs), which have been associated with a variety of adverse health effects in humans and wildlife^75,76^. Among the known harmful PAHs, toluene is a neurotoxicant used in glue^77^, benzo[a]pyrene (B[a]P) is both a neurotoxicant^78^ and a potent carcinogen^79,80^, while naphthalene can cause hemolytic anemia and is the active substance in mothballs^81^. Several of the 16 PAHs identified by the US Environmental Protection Agency as priority pollutants are present in cigarette smoke, such as B[a]P, pyrene, and phenanthrene^82^, but possible relations between PAH exposure and AD remain largely unexplored^72^.

**Figure 2:**
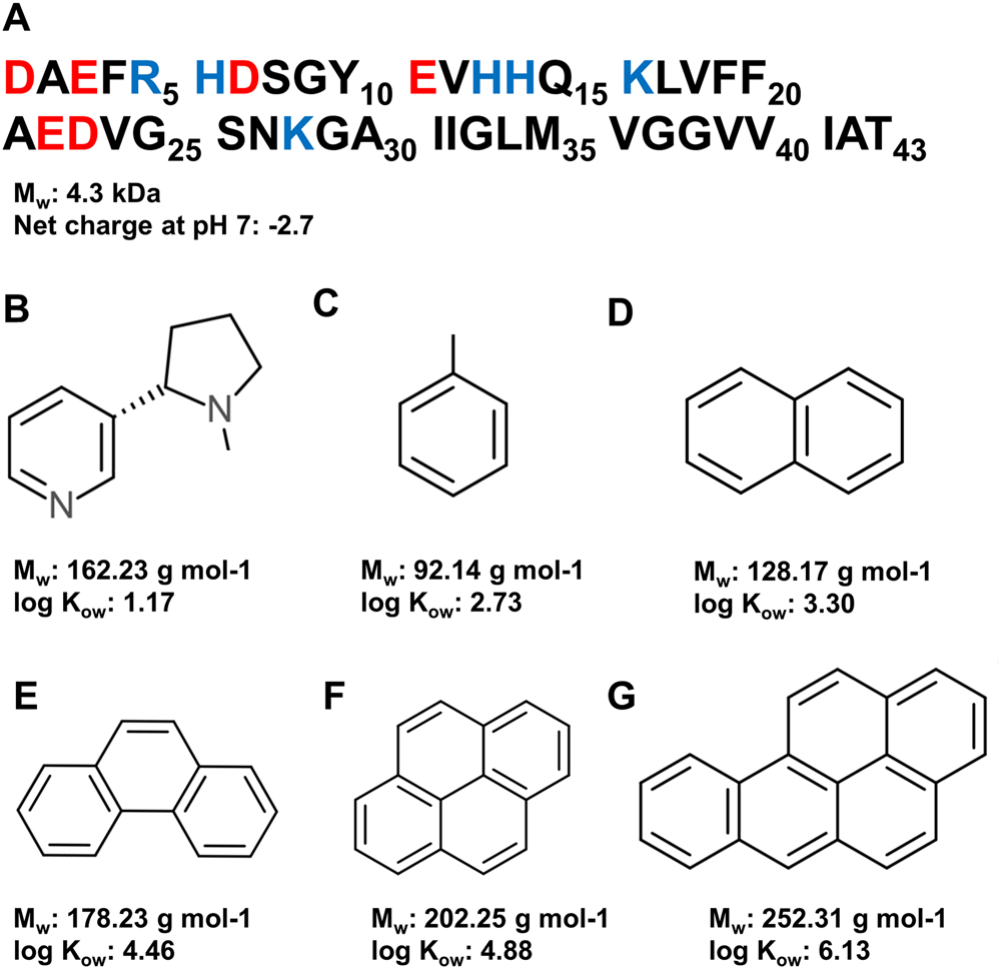
Primary sequence of the Aβ(1–43) peptide (**A**) together with chemical structures for (-)- nicotine (**B**) and the studied (poly)aromatic hydrocarbons, i.e. Toluene (**C**), Naphthalene (**D**), Phenanthrene (**E**), Pyrene (**F**), and Benzo[a]pyrene (**G**).

In this study, we monitored *in vitro* how a variety of substances in cigarette smoke affect Aβ aggregation and fibrillation, at a molecular level, using the Aβ(1–40) variant as a model peptide (from now on Aβ_40_). Biophysical techniques including nuclear magnetic resonance (NMR) spectroscopy, atomic force microscopy (AFM) imaging, ThT fluorescence assays, and mass spectrometry (MS) were used to investigate interactions between Aβ and i) the alkaloid nicotine, ii) hydrocarbons with one to five aromatic rings (Fig. 2), i.e., toluene, naphthalene, phenanthrene, pyrene, and B[a]P, and iii) the metal ions Pb(II), Pb(IV), Cd(II) and Cr(III). Many of these compounds are associated with adverse biological effects on survival, growth, development, reproduction, metabolism, and tumor formation^75,79–81^, and neurotoxic effects in particular have been associated with exposures to toluene^77^, B[a]P^78^, and several of the metals found in cigarette smoke^54,83–86^. The results presented below show that some of these substances may also contribute to AD pathogenesis and progression, by modulating the Aβ fibril formation process or/and by inducing ROS damage.

## Results

### NMR spectroscopy

**Figure 3:**
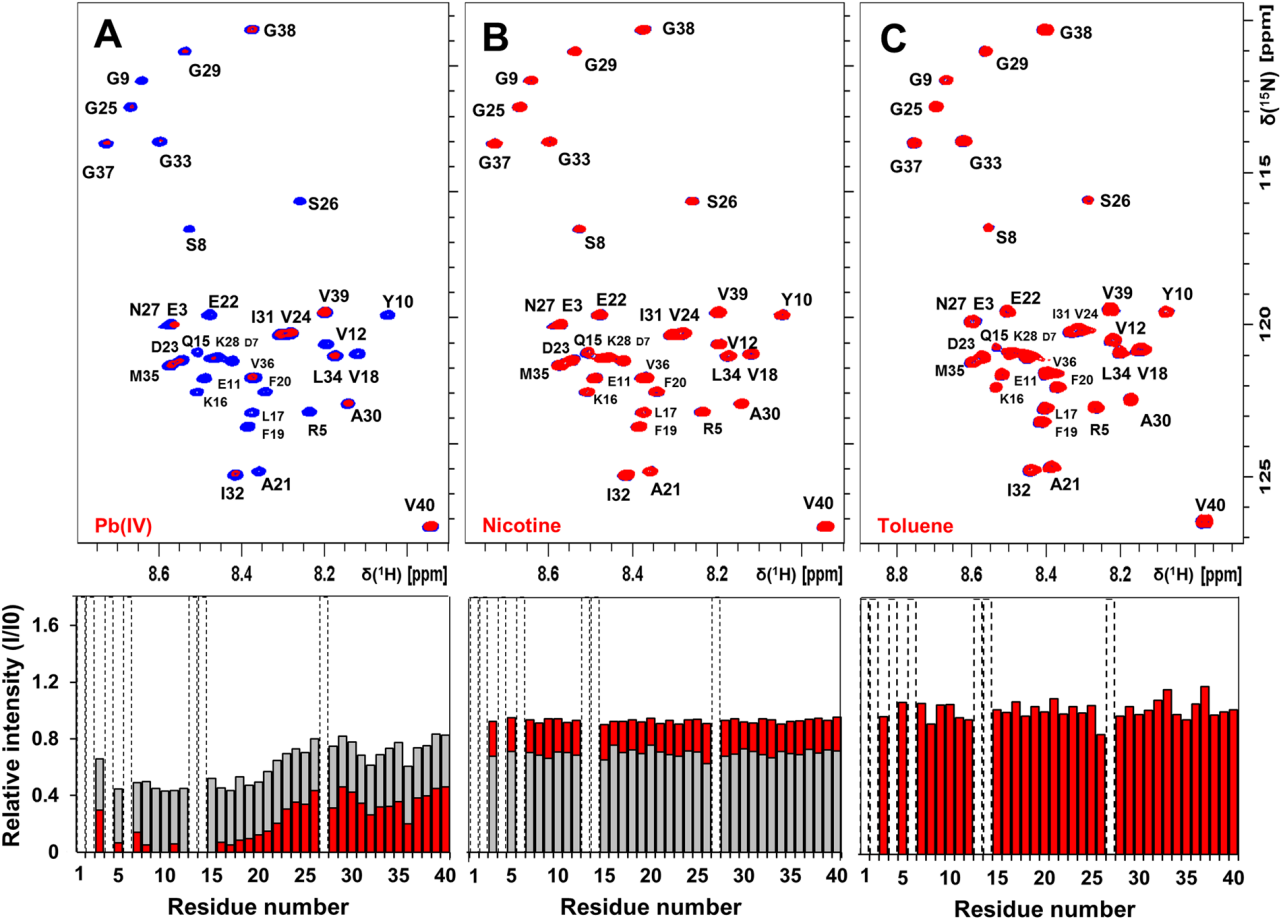
2D NMR spectra showing interaction effects of Pb(IV) ions, nicotine, and toluene on the Aβ monomer. 2D ^1^H,^15^N-HSQC spectra were recorded at +5°C for 84 µM (550 μl) monomeric Aβ(1–40) peptide in 20 mM sodium phosphate buffer, pH 7.35, before (blue) and after (red) addition of 504 μM Pb(IV) ions (**A**), 840 μM (-)- nicotine (**B**), or 20 μl toluene (**C**). Nicotine and Pb(IV) stock solutions were adjusted to pH 7.35 and titrated onto the sample, while toluene was added neat. The changes in amide crosspeak intensity are shown below the spectra, where the grey bars correspond to additions of 252 µM Pb(IV) ions (**A**) and 1680 µM (-)-nicotine (**B**). Dashed bars indicate amino acids that could not be observed due to spectral overlap or fast solvent exchange effects. In spectrum (**A**), specific interactions between Pb(IV) ions and the N-terminal part of the Aβ peptide are clearly visible.

**Figure 4:**
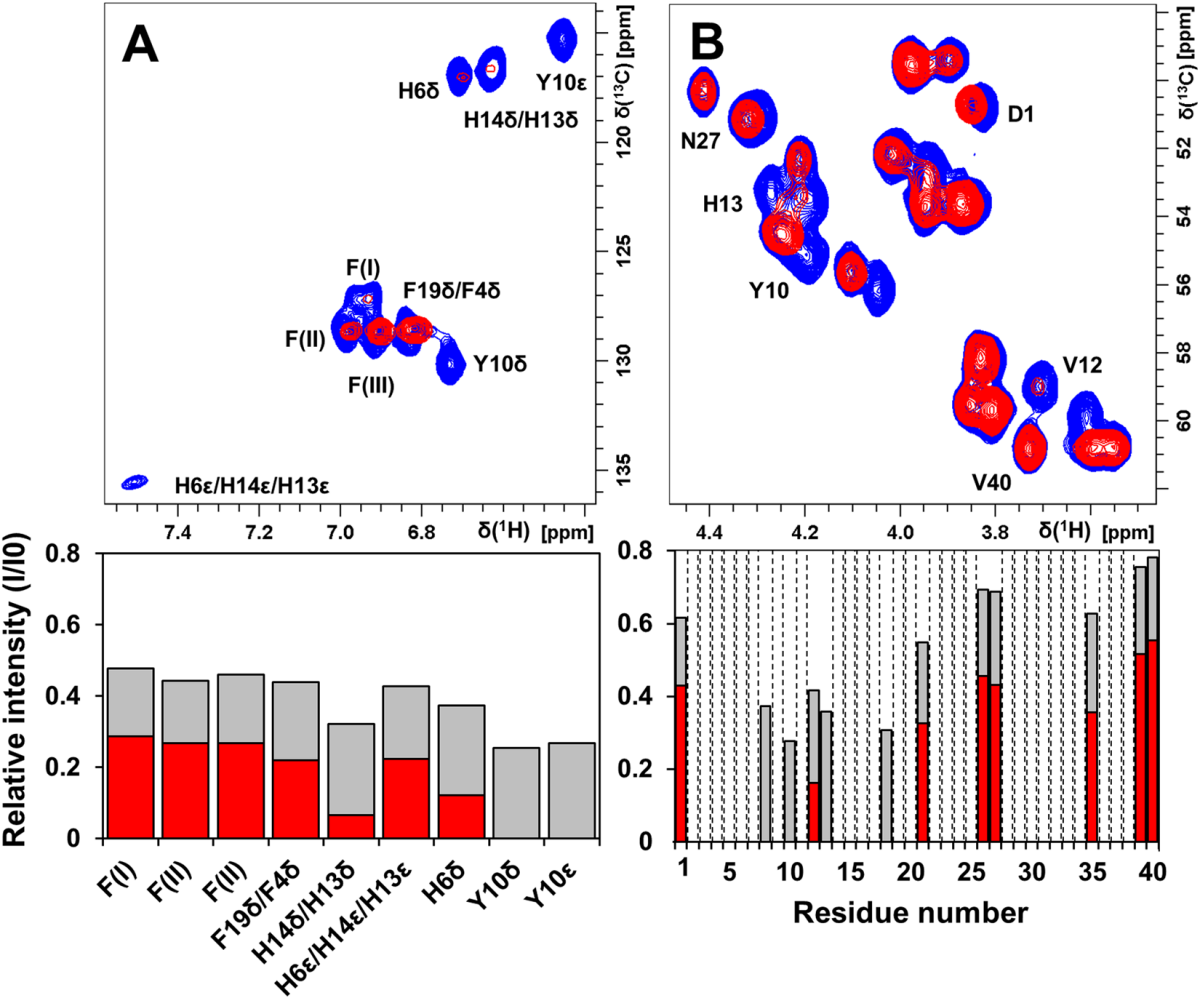
2D NMR spectra showing residue-specific interactions between the Aβ monomer and Pb(IV) ions. 2D ^1^H,^13^C-HSQC spectra were recorded at +5 °C for 84 µM monomeric ^13^C,^15^N-labeled Aβ(1–40) peptide in 20 mM sodium phosphate buffer, pH 7.35, before (blue) and after addition of 504 μM (red) or 252 µM (grey bars) Pb(IV) ions. Crosspeaks in the aromatic (**A**) and Cα-H (**B**) regions are shown, and the relative crosspeak signal intensities are presented below the spectra. Dashed bars indicate amino acids that could not be observed due to spectral overlap. Reduced signal intensities for N-terminal Aβ residues is clearly observed upon addition of Pb(IV) ions – residue Y10 is particularly affected.

NMR experiments were conducted to investigate possible molecular interactions between the monomeric Aβ_40_ peptide and the studied substances (Figs 3 and 4 and S1–S2). The finger-print region of a ^1^H,^15^N-HSQC spectrum of 84 µM (550 μl) monomeric unstructured ^15^N-labeled Aβ_40_ peptide is shown in Fig. 3, before and after addition of 504 µM Pb(IV) acetate (Fig. 3A), 840 µM nicotine (Fig. 3B), or 20 µL neat toluene (Fig. 3C). All compounds were titrated to the Aβ solution in small steps, starting at sub-stoichiometric ratios, but only spectra for larger additions are shown where the interaction effects (or lack thereof) are most pronounced. Pb(IV) ions clearly induce loss of signal intensity in the NMR amide crosspeaks corresponding to certain N-terminal Aβ_40_ residues, indicating specific binding to this part of the peptide (Fig. 3A). Similar effects were seen in the ^1^H,^13^C-HSQC spectra of ^13^C,^15^N-labeled Aβ_40_, where addition of Pb(IV) ions reduces the intensity of the aromatic and Cα-H crosspeaks for Y10 and the three histidines H6, H13, and H14 (Fig. 4). The loss of signal intensity is particularly strong for the Y10 crosspeaks (Fig. 4A), suggesting that Y10 or/and possibly E11 (Fig. 3A) is involved in Pb(IV) coordination. Although the present NMR observations cannot be directly interpreted in terms of binding geometry, the observed loss of NMR signal for specific residues is arguably caused by intermediate chemical exchange on the NMR time-scale between a free and a metal-bound state of the Aβ_40_ peptide. The residues most affected by the Pb(IV) ions are likely the most specific and strongly binding ligands^62^.

Addition of nicotine does not selectively affect the intensity or position of any particular Aβ ^1^H,^15^N-HSQC crosspeak, indicating that there are no specific interactions between nicotine and the Aβ monomer (Fig. 3B). Similarly, the metal ions Pb(II), Cd(II), and Cr(III) do not induce any specific changes in the Aβ ^1^H,^15^N-HSQC spectrum, indicating they have no strong specific binding to monomeric Aβ (Supplementary Fig. S1). Additions of naphthalene, phenanthrene, pyrene, and B[a]P induced small changes in crosspeak intensities and chemical shifts (Fig. S2). All these hydrocarbons were however dissolved in DMSO, and the small crosspeak effects observed are virtually identical to the spectral effects induced by small amounts of pure DMSO additions onto Aβ (Supplementary Fig. S1). Toluene, which was added neat, induced minor non-specific chemical shift changes in the crosspeak positions (Fig. 3C). We conclude that none of the studied hydrocarbons display any specific molecular interactions with monomeric Aβ.

### ThT fluorescence kinetics

**Figure 5:**
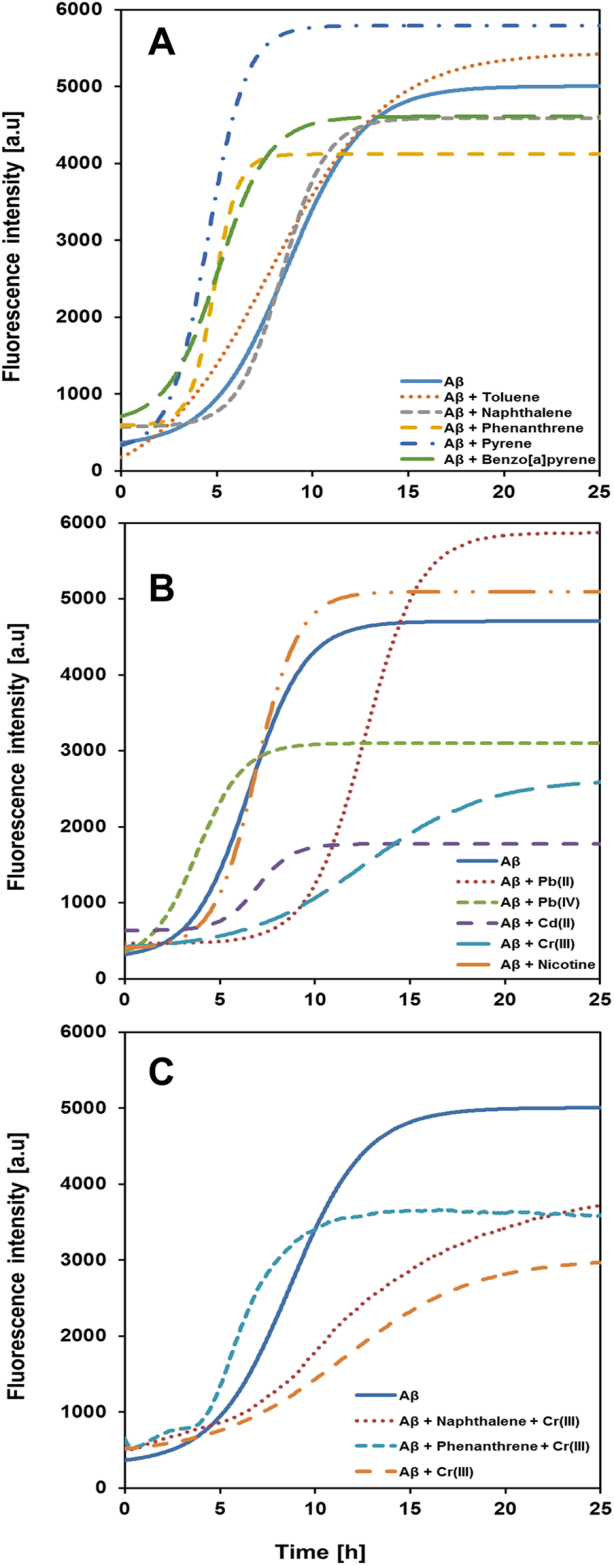
Aggregation kinetics of Aβ(1–40) peptides in the absence and presence of the studied compounds. (**A**) aromatic hydrocarbons, (**B**) metal ions and (-)-nicotine, and (**C**) combined aromatic hydrocarbons and Cr(III) ions. The Aβ kinetics was monitored by recording Thioflavin T (ThT) fluorescence intensity in 20 mM sodium phosphate buffer, pH 7.35, at +37 °C under quiescent conditions (ratio 1:10, Aβ:substance). In the figures averaged curves from five or six replicates are shown.

Figures 5 and S3 show ThT fluorescence intensity curves for Aβ_40_ aggregation kinetics that monitor the formation of amyloid in the presence of the studied substances. These kinetic curves have a general sigmoidal shape, and the kinetic parameters τ_½_ and r_max_ obtained from curve-fitting to Eq. 1 (in Materials and Methods) are shown in Table 1. The hydrocarbons toluene and naphthalene have no significant effect on the aggregation kinetics, while the larger phenanthrene, pyrene, and B[a]P molecules all increase the Aβ aggregation rate. The metal ions Cr(III) and Pb(II) significantly slow down the Aβ aggregation kinetics, which however is promoted in the presence of Pb(IV) ions. Cd(II) ions as well as nicotine appear to induce slightly slower aggregation, but the differences are too small to be significant (Table 1). The Aβ aggregation in the presence of both Cr(III) ions and naphthalene, or both Cr(III) ions and phenanthrene, is faster than with Cr(III) ions alone, yet slower than with naphthalene or phenanthrene alone (Table 1). Thus, the aggregation-promoting effects of the hydrocarbons – especially phenanthrene – seem to counteract the aggregation-retarding effect of the Cr(III) ions. The ThT fluorescence intensity at the endpoint plateau phase arguably corresponds to the amount of ThT-active (amyloid) aggregates being present. Following this assumption, it seems that the organic compounds have no significant effect on the amount of amyloid formed, while Cd(II), Cr(III), and Pb(IV) ions appear to induce a lower amount of ThT-active amyloid material at the end of the reaction (Table 1).

**Table 1:** Kinetic parameters for Aβ fibril formation.

	τ_½_ [h]	r_max_ [h^−1^]	ThT end point amplitude fluorescence [a.u]
Aβ in buffer	6.5 ± 0.9	1.0 ± 0.5	4200 ± 1100
Aβ in buffer + DMSO*	8.6 ± 0.6	0.6 ± 0.2	4500 ± 870
Aβ + Toluene	8.1 ± 1.0	0.6 ± 0.1	4800 ± 490
Aβ + Naphthalene*	8.4 ± 0.8	0.9 ± 0.2	4100 ± 600
Aβ + Phenanthrene*	4.9 ± 0.3	1.6 ± 0.6	3700 ± 460
Aβ + Pyrene*	4.6 ± 0.3	1.2 ± 0.4	5200 ± 390
Aβ + Benzo[a]pyrene*	5.1 ± 0.7	0.8 ± 0.3	4170 ± 900
Aβ + Pb(II)	15.2 ± 4.3	0.8 ± 0.4	6600 ± 1300
Aβ + Cd(II)	7.1 ± 0.5	1.0 ± 0.1	1200 ± 150
Aβ + Cr(III)	15.0 ± 4.0	0.4 ± 0.2	2700 ± 150
Aβ + Pb(IV)	3.9 ± 0.2	1.1 ± 0.4	2900 ± 140
Aβ + Nicotine	6.8 ± 0.7	1.1 ± 0.1	4500 ± 270
Aβ + Naphthalene + Cr(III)*	11.8 ± 1.4	0.3 ± 0.1	3800 ± 410
Aβ + Phenanthrene + Cr(III)*	6.2 ± 0.6	0.9 ± 0.1	3000 ± 210

ThT fluorescence data reflecting Aβ amyloid formation was recorded in the presence of metal ions, nicotine, and polycyclic hydrocarbons. Aggregation halftimes (τ_½_), maximum growth rates (r_max_), and ThT end point fluorescence amplitudes were derived from sigmoidal curve-fitting to Eq. 1. The samples marked with an asterisk (*) were measured with small amounts of DMSO present.

### AFM imaging

**Figure 6:**
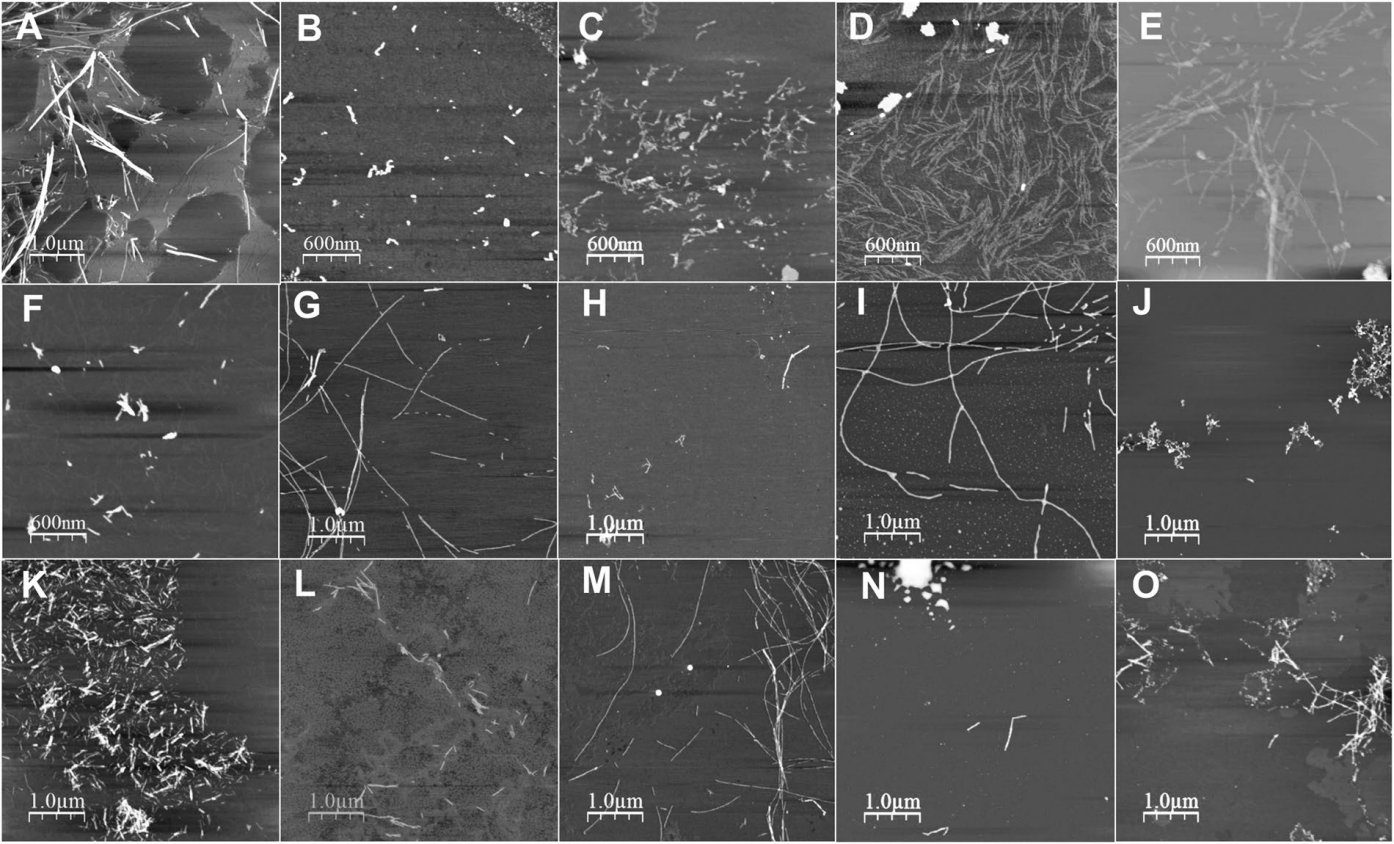
Solid state AFM images of Aβ aggregates. AFM images were recorded for 100 µM Aβ(1–40) peptides in 20 mM sodium phosphate buffer pH 7.35 with various combinations of 1000 µM aromatic hydrocarbons, metal ions, and nicotine, incubated for 6 hours (200 rpm) at +37 °C in Eppendorf tubes before dilution on a mica surface. (**A**) Aβ control in buffer and DMSO; (**B**) Aβ and toluene; (**C**) Aβ and naphthalene; (**D**) Aβ and phenanthrene; (**E**) Aβ and pyrene; (**F**) Aβ and B[a]P; (**G**) Aβ control in buffer; (**H**) Aβ and Cd(II) ions; (**I**) Aβ and Cr(III) ions; (**J**) Aβ and Pb(II) ions; (**K**) Aβ and Pb(IV) ions; (**L**) Aβ and Pb(IV) ions; (**M**) Aβ and (-)-nicotine; (**N**) Aβ, Cr(III) ions and naphthalene; (**O**) Aβ, Cr(III) ions and phenanthrene.

AFM images were recorded for 100 µM Aβ_40_ peptides incubated for 6 hours with or without added substances. Proper elongated Aβ fibrils are formed for Aβ_40_ alone under the conditions used (Fig. 6G), and also in the presence of added DMSO (Fig. 6A), nicotine (Fig. 6M), Cr(III) ions (Fig. 6I), or pyrene (Fig. 6E). Incubation with the other hydrocarbons inhibits proper fibril formation – instead smaller fibril fragments or amorphous aggregates are produced (Fig. 6B–D,F). Amorphous aggregates are also observed for Aβ incubated with the metal ions Cd(II), Pb(II), and Pb(IV) (Fig. 6H–I, K–L). Incubation in mixtures of Cr(III) ions and naphthalene (Fig. 6N), or Cr(III) ions and phenanthrene (Fig. 6O), produces combinations of amorphous aggregates and shorter fibrils.

### Mass spectrometry

**Figure 7:**
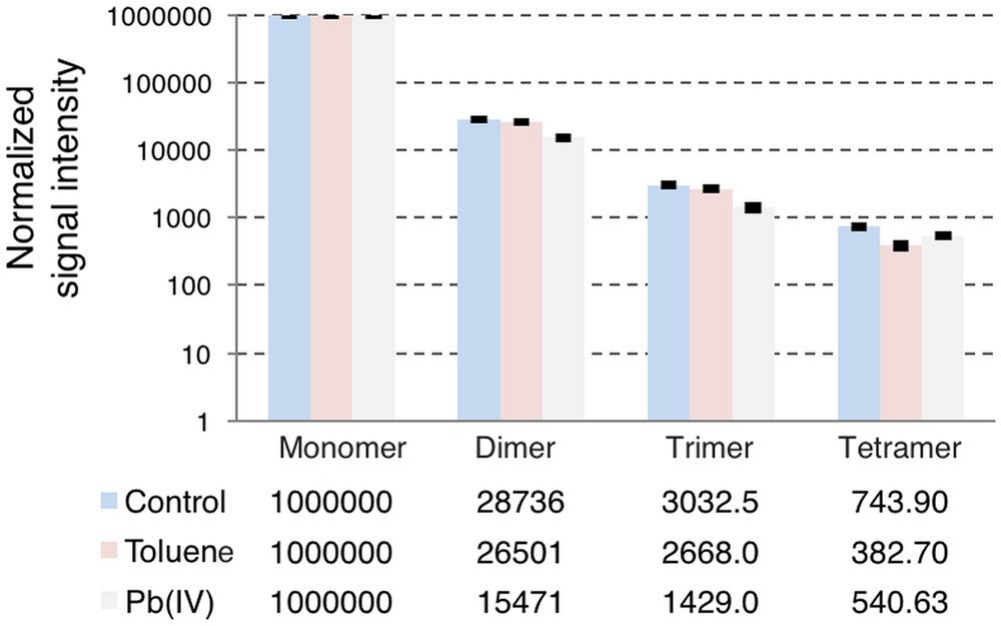
Relative populations of Aβ monomeric and oligomeric states. High resolution mass spectrometry together with soft sample ionization was used to measure the relative amounts of monomer, dimer, trimer, and tetramer populations for 20 μM Aβ(1–40) peptide prepared with and without the presence of 1:1 toluene or 1:1 Pb(IV) acetate. Pb(IV) ions reduce the relative amounts of dimers and trimers, while toluene mainly affects the tetramer population. The staple bars show average values for three replicates, while the error bars show two standard deviations.

Using protocols for soft ionization developed during the last decade, the recorded MS spectra provide information on non-covalent aggregated states of the Aβ_40_ peptide (i.e., from monomers up to dodecamers under favourable conditions)^87–89^. Under the experimental conditions used, we observe that toluene and Pb(IV) ions induce clear effects on the Aβ oligomer distribution up to tetramers (Figs 7 and S4). The Aβ sample freshly prepared in ammonium acetate solution is seen to be in equilibrium between the dominant monomeric form and smaller fractions of soluble dimers, trimers, and tetramers. The sample prepared in presence of toluene (1:1 Aβ:toluene ratio) shows a lower relative amount of tetramers (Fig. 7). The sample prepared in the presence of Pb(IV) ions (1:1 Aβ:Pb ratio) shows decreased relative amounts of dimers and trimers, but not tetramers. The total MS signal was lower for the sample containing Pb(IV) ions, which could indicate that addition of Pb(IV) ions immediately shifts the equilibrium towards higher molecular weight species. The presence of free metal ions in the sample should however also result in lower ionization efficiency for the Aβ peptide, which would reduce the signal intensity.

We furthermore observed that the presence of Pb(IV), a known oxidative agent^90,91^, induced significant oxidation of the M35 residue: 6% of the Aβ_40_ monomers were oxidized in the Pb(IV) sample compared to 2% in the control and toluene samples (Supplementary Fig. S4).

## Discussion

### Nicotine

Nicotine is the most abundant alkaloid in tobacco leaves and one of the most addictive substances known^92^. At high doses nicotine is toxic and even lethal^93^. Smoking a cigarette yields about 1–2 mg of absorbed nicotine, which readily is transported to the brain where it acts as an agonist on nicotinic acetylcholine receptors in the central nervous system^94^. A few hours after exposure, the absorbed nicotine is metabolized into various forms of cotinine^94^.

Our current results show that even at a 10:1 ratio, nicotine does not interact with the Aβ_40_ monomer; does not affect Aβ aggregation; and does not alter Aβ fibril morphology. We conclude that nicotine has no significant effect on Aβ and its aggregation pathway, which is in line with certain previous observations^95,96^. As AD is considered to be strongly related to amyloid aggregation^12,97,98^, a substance may affect an individual’s Aβ amyloid burden in any of three principal ways: by modulating the Aβ aggregation process, by binding to Aβ aggregates and thereby altering their biological effects, or by affecting Aβ production, degradation, or/and localization. For nicotine, our current results rule out the first two mechanisms, but effects on AD via the third mechanism remains a possibility that should be further explored. In addition, nicotine has been suggested to affect tau phosphorylation^99^, and to attenuate Aβ neurotoxicity by regulating metal homeostasis^100^.

### Metals

With one cigarette containing up to 1.5 μg Cd, 0.5 μg Cr, and 1.2 μg Pb, it has been shown that smokers have higher blood concentrations of these metals than nonsmokers^101^. Our results show that Cr(III), Cd(II), and Pb(II) ions display general and non-specific electrostatic interactions with Aβ_40_, and slow down the aggregation kinetics of the peptide, whereas Pb(IV) ions induce faster Aβ aggregation and display a specific binding mode to the Aβ monomer. Thus, together with e.g. Cu(II), Zn(II), Fe(II), and Mn(II) ions^59,102^, Pb(IV) appears to belong to a family of metal ions displaying specific and relatively strong binding interactions with the Aβ peptide. Addition of Pb(IV) ions to Aβ_40_ strongly affects the NMR signals of H6, Y10, E11, H13, and H14, indicating these residues as likely binding ligands. Previous studies on Aβ interactions with e.g. Cu(II) and Zn(II) ions indicate that multiple binding conformations likely co-exist^59^, and that the Aβ binding properties change at lower pH when the histidines become protonated^59,61,63^. Here, Y10 is strongly affected by Pb(IV), suggesting it is a major binding ligand (Figs 3A and 4). Pb(IV) ions are therefore likely to have a different binding coordination to Aβ than e.g. Cu(II) and Zn(II) ions, which are coordinated mainly by the three N-terminal histidines (H6, H13, and H14) together with the D1 residue^103^. This finding shows that Aβ metal-binding may be more complex and varied than previously thought, and should be further investigated.

While normal Aβ fibrils form in the presence of Cr(III) ions, our AFM images show formation of amorphous Aβ aggregates in the presence of Cd(II), Pb(II), and Pb(IV) ions. Thus, even though their binding is non-specific and weak, Cd(II) and Pb(II) ions are capable of altering the Aβ aggregation pathway *in vitro*. This is in line with previous work showing that Ca(II) ions promote Aβ fibrillation, even though Ca(II) ions show no specific interaction with the Aβ monomer^104^. However, *in vivo* mainly metal ions with relatively strong and specific binding to Aβ, and which are present in the brain in reasonable concentrations, are believed to modulate the Aβ aggregation process. Typical examples are Cu(II) and Zn(II) ions, which both have strong affinities for the Aβ monomer, and which both are released in high local concentrations from neuronal synapses^105^. As Cd(II), Cr(III), and Pb(II) ions display weak binding to Aβ and are present only as trace contaminants in human fluids, it appears unlikely that these metal ions would have a strong effect on Aβ aggregation *in vivo*, at least on their own.

Pb(IV) ions, on the other hand, display a stronger and specific binding to monomeric Aβ_40_, and the oxidation of Aβ residue M35 by Pb(IV) ions show that these ions can act as oxidizing agents and thus produce harmful ROS when bound to the peptide. Although Cu, Fe, and Zn are the main metals found in the amyloid brain plaques in AD patients^21,56–59,106^, lower concentrations of other metals including Pb have also been observed^58^. These lower Pb levels may not be surprising, as about 95% of the Pb that enters the adult body accumulates in the skeleton where Pb(II) replaces Ca(II) in the bone apatite^107^. Yet, the minor fraction of Pb that enters the brain appears to have biological impact, as Pb exposure has been correlated with a variety of adverse effects on neuronal formation, neurotransmission, and cognitive function^4,86,108^. Given our NMR results, it can be speculated that the Pb previously observed in AD brain plaques c Pb(IV). Future research will hopefully be able to shed more light on the oxidation states of the Pb that is distributed in different body tissues and fluids. It has previously been suggested that the Cu and Fe ions bound to Aβ plaques may generate damaging ROS via Fenton chemistry^59,64,109^, which would contribute to the neuroinflammation observed in AD patients^67^. In such scenarios the less reactive Zn(II) ion may protect nerve cells from radical damage by competing away harmful Cu and Fe ions from the Aβ metal binding sites^59^. As generation of oxygen radicals is one of the main mechanisms of Pb toxicity^90,91^, it appears very likely that the Pb bound to Aβ plaques will produce harmful ROS in the areas around these plaques. Given the local accumulation of Pb in these plaques, the resulting ROS damage likely affects AD pathology more than the aggregation-modulating properties of Pb(IV) ions, as we are not aware of any evidence for co-localization of elevated Pb and Aβ aggregation pathways. Nevertheless, together with previous observations that Pb exposure induces elevated Aβ levels in rats^110^, increased Aβ plaque formation in monkeys^111^, and enhanced tau production and phosphorylation in both mice and monkeys^112,113^, our current results that Pb(IV) ions display specific binding to the Aβ peptide, alter its aggregation process, and cause oxidative effects in the presence of Aβ, clearly show that Pb ions can modulate the Aβ amyloid cascade events that are associated with AD.

### Hydrocarbons

Cigarette smoke contains a wide range of aromatic hydrocarbons that deposit and accumulate in the lungs as cigarette tar. Some of this tar is absorbed by the body, and the hydrophobic hydrocarbons can permeate the lipophilic blood-brain barrier membrane, allowing transport into the brain^114^. Within a few days, absorbed aromatic hydrocarbons are typically metabolized in multistep reactions into e.g. epoxides and polar hydroxyl-derivatives, leading to end products that the body readily can excrete^115^.

Our ThT fluorescence results show that addition of phenanthrene, pyrene, or B[a]P increase the Aβ_40_ aggregation rate, which remains largely unaltered when toluene or naphthalene is added (Fig. 5; Table 1). The AFM images show that toluene, naphthalene, and B[a]P induce formation of amorphous Aβ aggregates, while relatively unaltered fibrils are formed in the presence of phenanthrene and pyrene (Fig. 6). Thus, there is no clear correlation between the aggregation kinetics and the aggregation products formed. Instead, the kinetic monitoring and the AFM images provide complementary information. For example, even though pyrene promotes and Cr(III) ions retard Aβ aggregation (Fig. 5; Table 1), proper fibrils are formed in the presence of both compounds (Fig. 6). Cd(II) ions also retard Aβ aggregation, but here amorphous aggregates are formed instead of fibrils (Figs 5–6; Table 1).

The NMR data reveal that there is no strong interaction between the monomeric Aβ_40_ peptide and any of the studied hydrocarbons, suggesting that hydrocarbons are unlikely to initiate (seed) Aβ aggregation. Taken together, these results can be explained in terms of hydrophobicity. The amphiphilic Aβ monomers are not attractive binding partners for the hydrophobic hydrocarbons. Aβ aggregation is however driven by hydrophobic interactions, where the oligomers formed are considered to be micelle-like entities with a hydrophobic core^11^. Such a core would readily attract aromatic molecules, arguably leading to formation of micelle-like Aβ-PAH co-aggregates (depending on the concentrations and Aβ/PAH ratios involved), thereby inducing deviations from the fibril-forming aggregation pathway. Our mass spectrometry data support such a scenario, as hydrophobic toluene was found to affect larger Aβ tetramers but not the smaller dimers or trimers (Fig. 7). The Pb(IV) ions, on the other hand, affected the dimer and trimer populations more than the tetramers (Fig. 7). This suggests that electrostatic interactions may be more important for the first steps of Aβ aggregation, and hydrophobic effects more important for larger oligomer formation. Such a scenario is consistent with nicotine not affecting Aβ aggregation at all, as nicotine is a hydrophilic weak organic base that is only slightly positively charged at neutral pH (its pyrrolidino N has a pKa around 8^116^). Elucidating the forces governing different stages of Aβ aggregation is important not only for understanding the amyloid formation process as such^117^, but may also help in designing interacting molecules (drugs) that can selectively target certain (toxic) aggregation states^118^. Thus, this difference in the interaction of Aβ with metal ions and PAHs respectively should be further explored, together with the question how promoting or retarding Aβ aggregation – into amyloid fibrils or amorphous material – affects AD progression. Future research on the molecular details of the likely toxic Aβ oligomers, where the structures remain poorly understood^10,11^, may also be able to explain why the more hydrophobic three-, four-, and five-ring molecules affect the Aβ aggregation kinetics more than the one- and two-ring compounds (Fig. 5, Table 1).

### Combined effects

Toxic or biological effects of chemical substances are often investigated one substance at a time, even though real-life exposure scenarios typically involve exposure to multiple chemicals. The thousands of different compounds in cigarette smoke is a case in point. For PAHs it is well known that synergistic effects can make them far more toxic in combination than alone^119^. For AD, a recent study showed that rats exposed to a mixture of Pb, Cd, and As produced greater increases in Aβ levels and cognitive impairment related to oxidative stress and inflammation than the sum of the individual metals^120^. This could be due to synergistic effects, or due to overloaded protective mechanisms. Our current measurements indicate that the retarding effects of Cr(III) ions and the promoting effects of naphthalene/phenanthrene on Aβ peptide aggregation kinetics counteract each other when both substances are present (Fig. 5C). Thus, given the multitude of compounds present in cigarette smoke, the overall effect of cigarette smoking on Aβ aggregation and AD pathology will be difficult to elucidate at a molecular level, at least until precise neurotoxic mechanisms underlying AD have been firmly established.

Due to the requirements of the analytical techniques used, the *in vitro* experiments were carried out with somewhat higher reagent concentrations than typically found in the human body. The Aβ_40_ concentrations in our experiments were in the range 10–100 uM, while the Aβ_40_ concentration in cerebrospinal fluid (CSF) is in the range 14–23 ng/L (3–5 pM) for AD patients and 10–18 ng/L (2–4 pM) for healthy controls^121^. Aggregation of Aβ *in vivo* is however likely to take place in e.g. membrane environments, where the local Aβ concentration is higher. The cigarette smoke compounds were also investigated at higher concentrations than what is found *in vivo*, although for the studied interactions the Aβ:compound ratio is likely more important than the actual compound concentration. The superstoichiometric ratios required for the studied compounds to have significant effects indicate that they are not likely to be major *in vivo* modulators of Aβ aggregation on their own, but could be important factors in combination with other compounds. As concentrations of e.g. Cu, Fe, Pb, and Zn are elevated in amyloid brain plaques, the combined ROS generated by Cu, Fe, and Pb ions arguably contribute to the neuronal damage observed in AD, and as metal dyshomeostasis appears to be involved in AD pathology^59,71^, future research should elucidate how different combinations of metal ions affect the Aβ amyloid cascade events.

### Conclusions

Smoking is an established risk factor for Alzheimer’s disease and other neurodegenerative disorders. Here, five aromatic hydrocarbons and four metal ions present in cigarette smoke were found to affect the Aβ_40_ peptide aggregation process. Metal ions such as Pb(IV) appear to mainly affect formation of Aβ dimers and trimers, while hydrocarbons such as toluene appear to mainly affect larger oligomeric and hydrophobic forms such as tetramers. Some metal ions and hydrocarbons counteract each other’s overall effects. The uncharged and hydrophilic nicotine molecule has no direct effect on Aβ or its aggregation process. As Pb(IV) ions interacting with Aβ were found to act as oxidizing agents – likely harmful – the specific binding observed between Aβ and Pb(IV) ions warrant further investigation, particularly given that significant sources of Pb exposure remain a major problem worldwide and especially in developing countries^86^.

## Materials and Methods

### Sample preparation

Unlabeled or uniformly ^15^N- or^13^C,^15^N-labeled Aβ(1–40) peptides were bought lyophilized from AlexoTech AB (Umeå, Sweden). The peptide samples were freshly dissolved and prepared before the measurements, according to previously published protocols^61^. Liquid (-)-nicotine (Sigma-Aldrich) and acetate salts of Cd(II), Cr(III), Pb(II), and Pb(IV) (Sigma-Aldrich) were dissolved or diluted in 20 mM phosphate buffer. Naphthalene, phenanthrene, pyrene, and B[a]P were first dissolved in 50% DMSO and then diluted in 20 mM phosphate buffer. NaOH/HCl was used to adjust the pH of all stock solutions to 7.35.

### NMR spectroscopy

2D heteronuclear single quantum coherence (^1^H,^15^N-HSQC and ^1^H,^13^C-HSQC) NMR experiments were performed on Bruker Avance 500 MHz and 700 MHz spectrometers equipped with cryoprobes. Spectra of monomeric 84 μM Aβ_40_ peptides uniformly labeled with ^13^C or/and ^15^N isotopes were recorded at + 5 °C in 20 mM sodium phosphate buffer pH 7.35 with 10% D_2_O. The studied cigarette smoke substances were titrated to the Aβ sample in small steps to final Aβ:substance molar ratios above 1:10. The Aβ_40_ HSQC crosspeak assignment is known from previous work^122^. All data was processed with the Topspin version 3.2 software and referenced to the ^1^H signal of TSP.

### AFM imaging

Solid state AFM images were recorded using a Bruker’s Scan Asyst (Bruker Corp., USA) unit operating in peak-force mode or tapping mode with a resolution of either 256 × 256 or 1024 × 1024 pixels. Solutions of 100 μM Aβ_40_ peptide in 20 mM sodium phosphate buffer at pH 7.35 were incubated at +37 °C in Eppendorf tubes at 200 rpm for 6 hours, either in absence (control) or in presence of one or two of the cigarette smoke substances (1000 μM additions). The incubated samples were then diluted and applied on freshly cleaved mica substrates. After 20 minutes the mica substrates were three times washed with distilled water and left to air-dry.

### ThT fluorescence

A 96-well FLUOstar Omega plate reader (BMG LABTECH, Germany) was used to record fluorescence spectra (excitation 440 nm; emission 480 nm) every three minutes for samples containing 10–20 μM Aβ_40_ peptides, 40 μM ThT dye, and 100–200 μM cigarette smoke substances together in 20 mM sodium phosphate buffer at pH 7.35. The measurements were running real-time for up to 48 hrs at +37 °C under quiescent conditions. The Aβ aggregation kinetic parameters τ^½^ and r_max_ were calculated by fitting five or six replicates per condition to a sigmoidal curve according to Eq.(1)^123^:

1 $$F(t)={F}_{0}+\frac{A}{1+\exp [{r}_{\max }({\tau }^{1/2}-t)]}$$

where F_0_ is the fluorescence intensity baseline, A is the fluorescence amplitude, r_max_ is the maximum growth rate, and τ_½_ is the time when half the monomer population is depleted.

### Mass spectrometry

Mass spectra of 20 μM Aβ_40_ peptide dissolved in 20 mM ammonium acetate buffer, pH 7.4, with and without addition of toluene and Pb(IV) acetate at 1:1 ratios were recorded three times each on a Synapt G2-Si high definition mass spectrometer (Waters corporation) equipped with a conventional ESI source operating in positive ion mode. Flow rate was 20 μl/min, capillary voltage 2.5 kV, cone voltage 40 V. Analysis was done in high-resolution mode (average resolution of 30 000) in the 500–4000 m/z range.

Data processing was done using the Proteowizard^124^, UniDec^125^, and mMass^126^ softwares. Peaks were identified by comparison between raw experimental data and generated theoretical peak lists, and by analysis of isotopic patterns (Supp. Fig. S4). All data were normalized to the +4 charge state signal of the Aβ monomer, to account for small deviations in concentration and ionization efficacy across samples.

### Data availability

All data generated or analysed during this study are included in this published article (and its Supplementary Information file).

## Electronic supplementary material

Supplementary Information
