# Supplementary material for: Alzheimer’s disease and cigarette smoke components: effects of nicotine, PAHs, and Cd(II), Cr(III), Pb(II), Pb(IV) ions on amyloid-β peptide aggregation
Source: Sci Rep. 2017 Oct 31;7:14423. doi: 10.1038/s41598-017-13759-5 (PMC5663743; doi:10.1038/s41598-017-13759-5)
Supplement: Supplementary file 1 — Supplementary Information [file 41598_2017_13759_MOESM1_ESM.pdf]

Supplementary Material

Alzheimer's disease and cigarette smoke components: effects of nicotine, PAHs, and Cd(II), Cr(III), Pb(II), Pb(IV) ions on amyloid-β peptide aggregation

Cecilia Wallin, Sabrina B. Sholts, Nicklas Österlund, Jinghui Luo, Jüri Jarvet,
Per M. Roos, Leopold Ilag, Astrid Gräslund, Sebastian K.T.S. Wärmländer

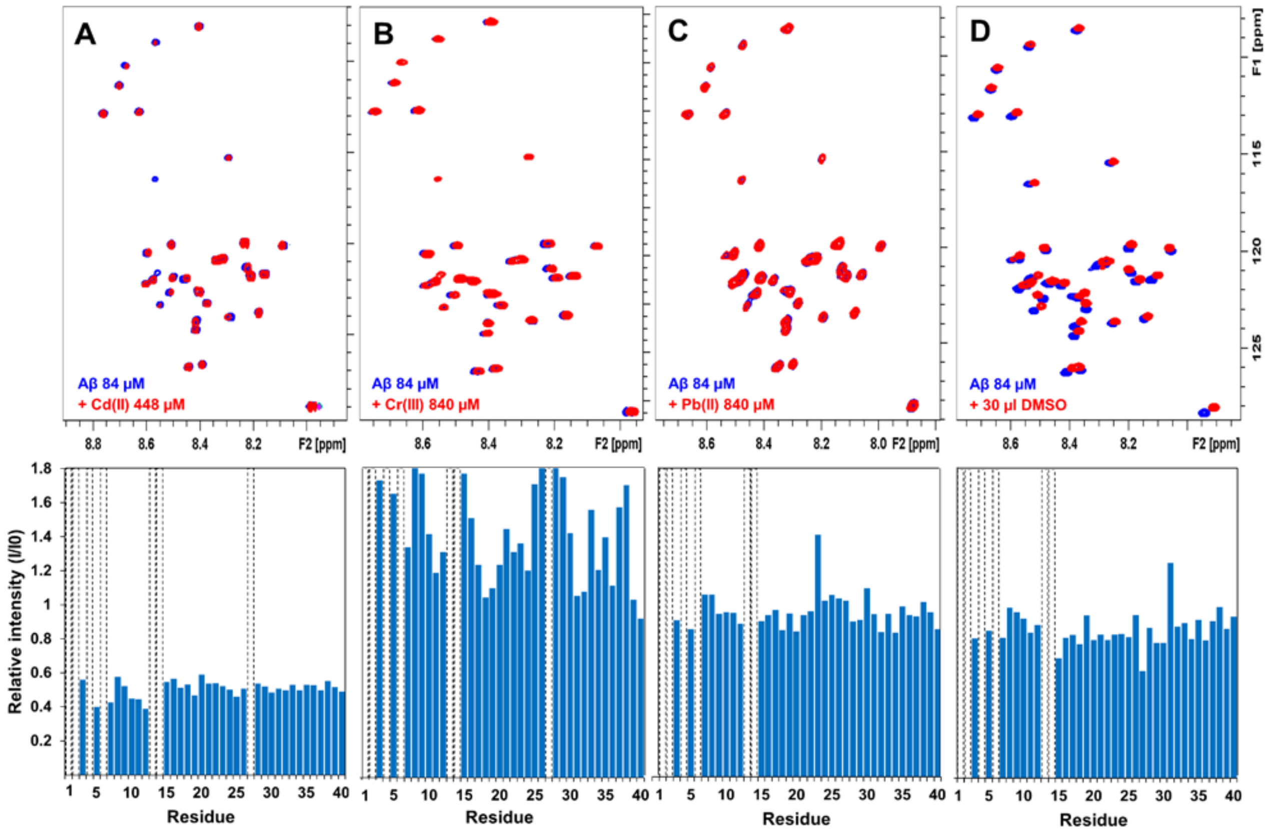

Supplementary Figure S1. NMR 2D ^{1}H, ^{15}N-HSQC spectra for 84 μM monomeric ^{15}N-labeled Aβ(1-40) peptides in 20 mM sodium phosphate buffer pH 7.35 at +5°C, recorded with a 500 MHz spectrometer equipped with a cryoprobe. The spectra show the Aβ(1-40) peptide before (blue) and after (red) addition of (A) 448 μM Cd(II) acetate; (B) 840 μM Cr(III) acetate; (C) 840 μM Pb(II) acetate; (D) 30 μl DMSO. The relative changes in amide crosspeak intensity are shown below the spectra. Dashed lines represent amino acids that were not observed in the spectra due to either spectral overlap or fast solvent exchange effects. No specific binding interactions were observed for the metal ions. The increased amide crosspeak intensities resulting from addition of Cr(III) acetate may relate to the Cr(III) ions shifting the Aβ peptide equilibrium towards a larger monomeric fraction. Figure (D) shows the characteristic chemical shifts induced by DMSO, which is relevant for interpreting Figure S2 (below).

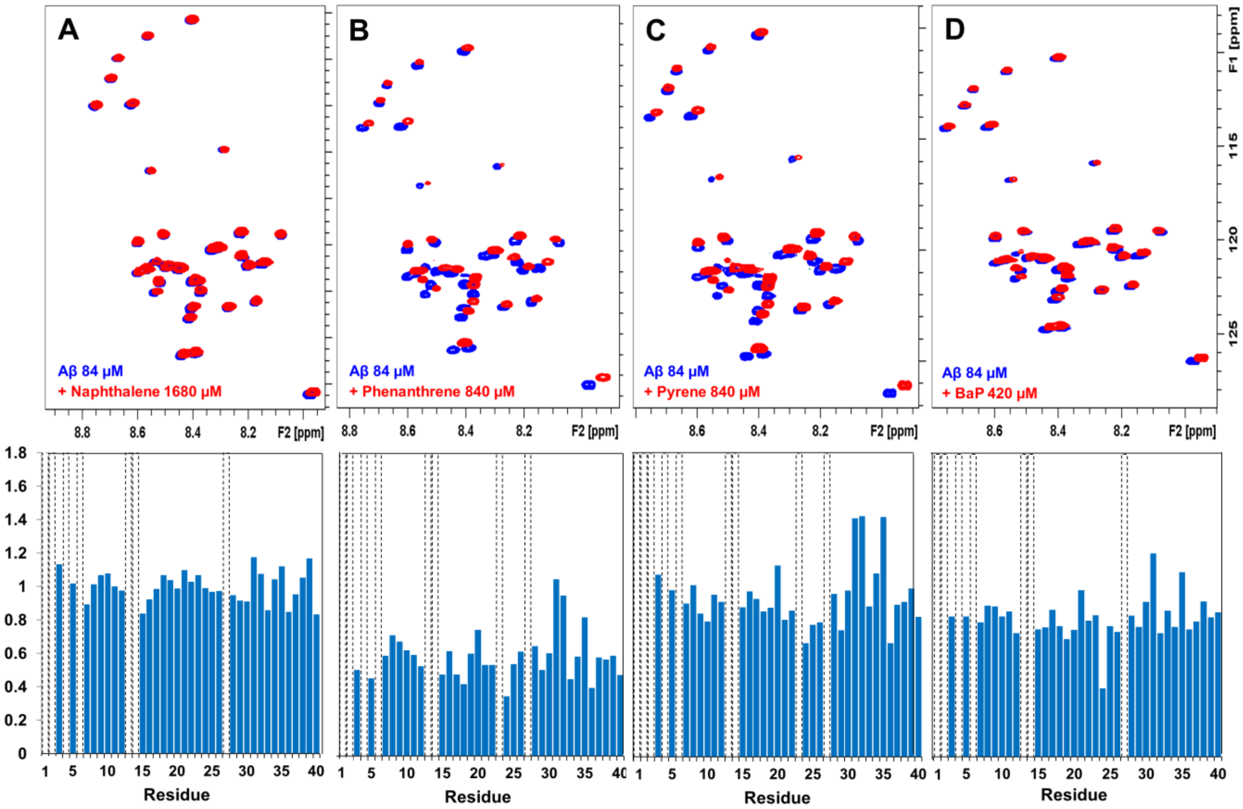

Supplementary Figure S2. NMR 2D ^{1}H, ^{15}N-HSQC spectra for 84 μM monomeric ^{15}N-labeled Aβ(1-40) peptides in 20 mM sodium phosphate buffer pH 7.35 at +5°C, recorded with a 500 MHz spectrometer equipped with a cryoprobe. The spectra show the Aβ(1-40) peptide before (blue) and after (red) addition of different hydrocarbons: (A) 1680 μM naphthalene; (B) 840 μM phenanthrene; (C) 840 μM pyrene; (D) 420 μM benzo[a]pyrene. The relative changes in amide crosspeak intensity are shown below the spectra. Dashed lines represent amino acids that were not observed in the spectra due to either spectral overlap or fast solvent exchange effects. No specific binding interactions were observed between Aβ and the hydrocarbons. The chemical shift differences observed in spectra (A) - (D) are induced by DMSO (cf Figure S1D).

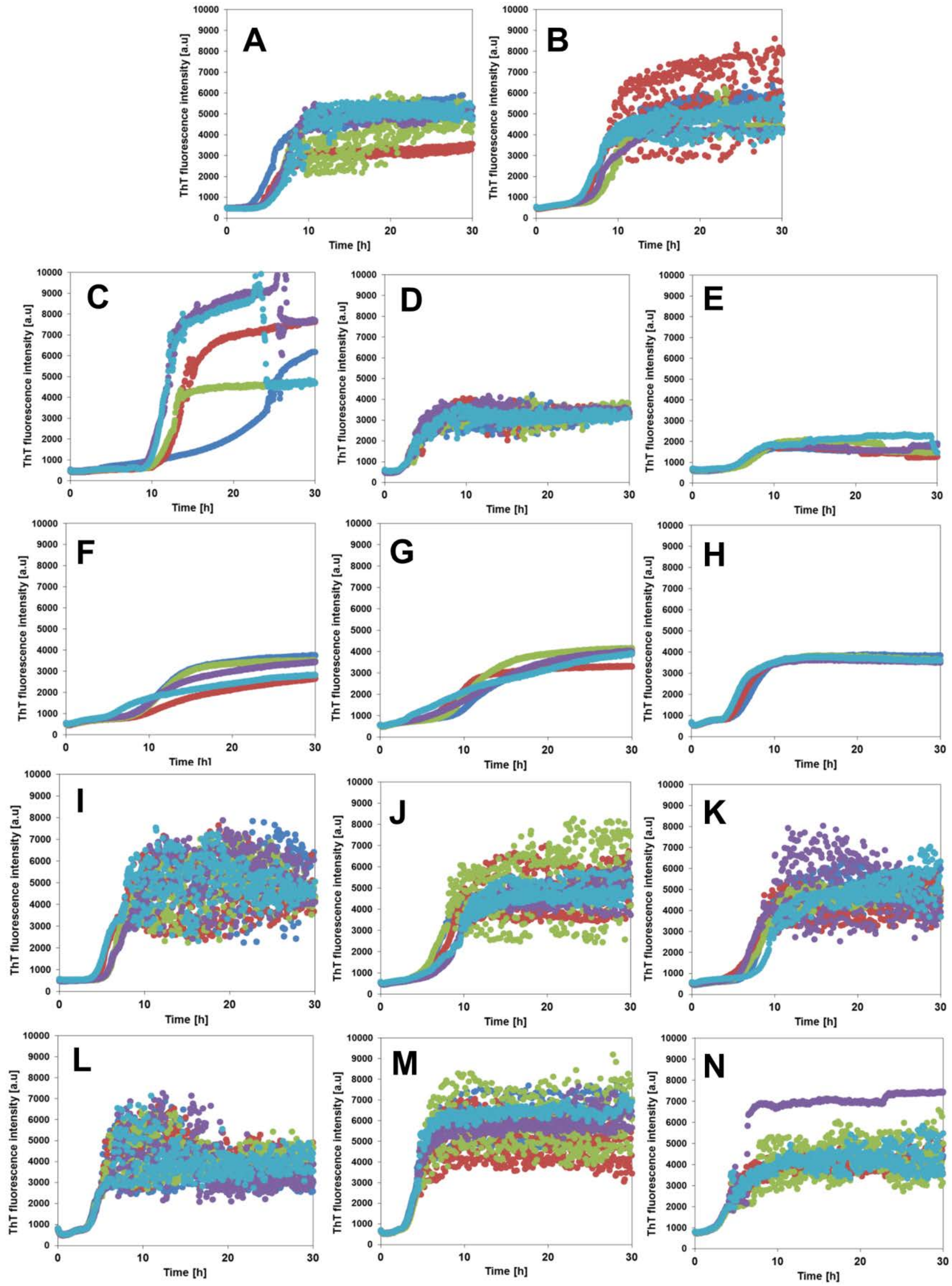

Supplementary Figure S3. Aggregation kinetics of Aβ(1-40) peptides in 20 mM sodium phosphate buffer, pH 7.35 at +37°C under quiescent conditions (ratio 1:10, Aβ:substance), monitored by Thioflavin T fluorescence. The raw data presented here was used to calculate the aggregation kinetics parameters shown in Figure 5 and Table 1 in the main manuscript. (A) Aβ in buffer; (B) Aβ in buffer + DMSO; (C) Aβ + Pb(II); (D) Aβ + Pb(IV; (E) Aβ + Cd(II); (F) Aβ + Cr(III); (G) Aβ + Cr(III) + Naphthalene; (H) Aβ + Cr(III) + Phenanthrene; (I) Aβ + Nicotine -/-; (J) Aβ + Toluene, (K) Aβ + Naphthalene; (L) Aβ + Phenanthrene; (M) Aβ + Pyrene; (N) Aβ + Benzo[a]pyrene.

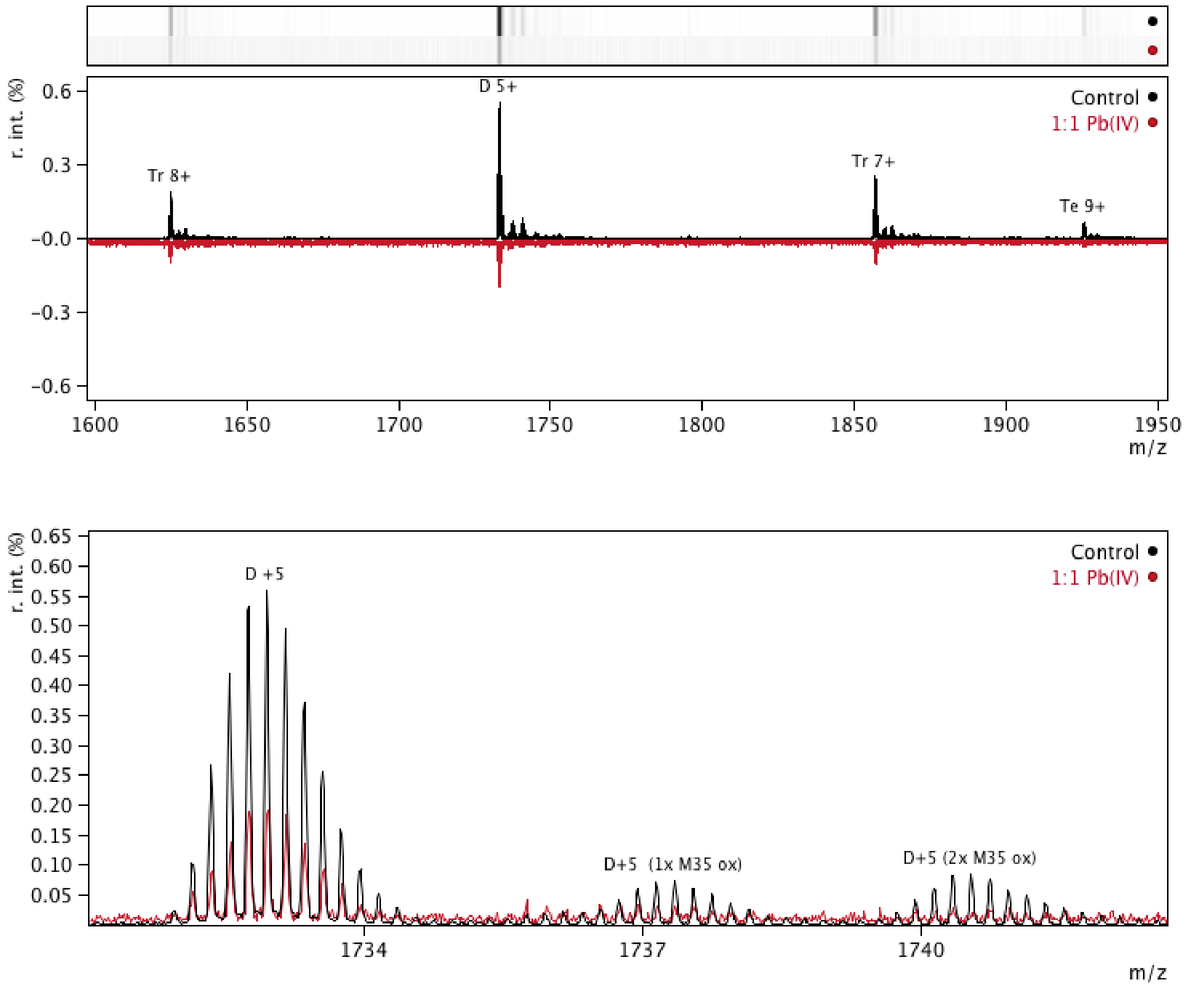

Supplementary Figure S4. Top: Typical oligomeric region in a mass spectrum of the Aβ(1-40) peptide alone (black trace) and in the presence of 1:1 Pb(IV) acetate (red trace), recorded in 20mM ammonium acetate buffer at pH 7.4. Peaks for trimer (+7/+8), dimer (+5), and tetramer (+9) are shown. All peaks are normalized to the +4 charge state of the Aβ monomer, and addition of Pb(IV) ions decreases the relative abundance of oligomers observed in the spectra. Bottom: Detailed view of the +5 charge state for the Aβ dimer. The well-resolved isotopic pattern allows exact identification of the oligomeric species. Aβ populations with oxidized methionine residues are also well separated. Average spectra from three replicates are shown.
